# Spontaneous Mesenteric Hematoma Following Severe Coughing in a Patient on Dual Antiplatelet Therapy: A Case Report

**DOI:** 10.7759/cureus.116672

**Published:** 2026-09-21

**Authors:** Saif Darwish, Manroop Minhas, Ryan Doyle, Monsurat Bonojo, Michael Zemaitis

**Affiliations:** 1 Clinical Medicine, American University of Antigua, St. John's, ATG; 2 Trauma Surgery, Richmond University Medical Center, New York, USA

**Keywords:** abdominal pain, bronchitis, conservative management, coughing, diverticulitis mimic, hematoma versus abscess, mesenteric hematoma, mesenteric hemorrhage, spontaneous hematoma, spontaneous mesenteric hematoma

## Abstract

Spontaneous mesenteric hematoma (SMH) is a rare cause of acute abdominal pain and may mimic more common intra-abdominal conditions, creating diagnostic challenges. A 67-year-old man with hypertension, hyperlipidemia, coronary artery disease status post four-vessel coronary artery bypass grafting, and chronic dual antiplatelet therapy with aspirin and clopidogrel presented with acute worsening lower abdominal pain. He reported a severe coughing episode during a recent bronchitis-like illness approximately two weeks before presentation, followed by lower abdominal pain in the same general region that persisted before acutely worsening on the day of admission. Initial computed tomography (CT) of the abdomen and pelvis demonstrated a large left lower abdominal collection adjacent to the sigmoid colon, raising concern for complicated diverticulitis with abscess formation or an infected hematoma. Repeat imaging favored a hematoma, and subsequent CT angiography demonstrated a stable collection without active contrast extravasation. Because the patient remained hemodynamically stable, with hemoglobin decreasing initially and subsequently stabilizing, and with no evidence of active bleeding, he was managed conservatively with close monitoring and supportive care. His abdominal pain improved, and he was discharged without surgical or interventional radiologic treatment. SMH should be considered in patients with atraumatic abdominal pain, particularly when imaging findings are atypical or mimic diverticulitis or other intra-abdominal pathology. In this case, the temporal relationship between severe coughing and subsequent localized abdominal pain raises the possibility that coughing-related increases in intra-abdominal pressure contributed to mesenteric vessel injury in the setting of dual antiplatelet therapy and underlying vascular risk factors. However, a direct causal relationship cannot be established. Early recognition and appropriate imaging can facilitate accurate diagnosis and support conservative management in hemodynamically stable patients without active bleeding.

## Introduction

Definition of mesenteric hematoma

Mesenteric hematoma is an uncommon condition characterized by bleeding within the mesentery, resulting in a localized blood collection between layers of mesenteric tissue. Mesenteric hematomas are most frequently associated with blunt abdominal trauma, postoperative complications, anticoagulation therapy, vascular abnormalities, or inflammatory conditions. Spontaneous mesenteric hematoma (SMH), defined as mesenteric hematoma occurring in the absence of trauma or other identifiable precipitating factors, remains a rare clinical entity and may present with nonspecific symptoms, including abdominal pain, nausea, vomiting, or signs of acute abdomen, making diagnosis challenging [1,2].

Epidemiology and rarity of SMH

SMH is rare, with few cases reported in the literature, mostly isolated case reports and small case series. Identified risk factors include anticoagulant use, coagulopathies, vasculitis, connective tissue disorders, pancreatitis, and rupture of visceral artery aneurysms [2,3]. Cases without identifiable causes are much less common and are often diagnosed only after excluding more common etiologies. Due to its rarity and variable presentation, SMH may initially mimic conditions such as diverticulitis, bowel ischemia, abscess formation, or intra-abdominal malignancy [3].

Common etiologies and risk factors

SMH is an uncommon but potentially life-threatening condition that most often occurs in the setting of an underlying vascular or hematologic abnormality. Reported risk factors include anticoagulant and antithrombotic therapy, inherited or acquired coagulopathies, vasculitis, connective tissue disorders such as Ehlers-Danlos syndrome, pancreatitis, malignancy, and rupture of visceral artery aneurysms or pseudoaneurysms [4-6]. Advanced age, hypertension, atherosclerotic disease, and other conditions associated with vascular fragility have also been reported and may contribute to an increased susceptibility to spontaneous bleeding [4-6]. Despite extensive evaluation, many cases remain idiopathic, with no identifiable bleeding source at the time of diagnosis or surgical exploration [4-6].

Anticoagulant therapy is among the most frequently reported associations in the literature; however, SMH has also been described in patients receiving antiplatelet therapy or without any identifiable medication-related risk factor [4,5,7]. The rarity of the condition, despite the widespread prevalence of hypertension, hyperlipidemia, cardiovascular disease, and antithrombotic use, suggests that additional precipitating factors may be necessary for hematoma formation. Proposed mechanisms include rupture of fragile mesenteric vessels, occult vascular abnormalities, or sudden increases in intra-abdominal or intravascular pressure that place stress on susceptible vessels [5-7].

Clinical presentation and diagnostic challenges

The clinical presentation of SMH is highly variable and often nonspecific, contributing to diagnostic delays. Patients most commonly present with acute or subacute abdominal pain, although nausea, vomiting, abdominal distention, gastrointestinal bleeding, anemia, hemodynamic instability, or signs of an acute abdomen may also occur, depending on the size and location of the hematoma. Laboratory findings are similarly nonspecific and may range from normal results to leukocytosis, elevated inflammatory markers, or progressive declines in hemoglobin concentration [5,8]. Because symptoms often overlap with those of more common intra-abdominal conditions, clinicians may initially mistake SMH for diverticulitis, mesenteric ischemia, bowel perforation, abscess, bowel obstruction, or intra-abdominal malignancy. In some cases, surrounding inflammatory changes on imaging may further obscure the diagnosis and raise concern for infectious or neoplastic processes. Given these diagnostic challenges, a high index of suspicion and timely cross-sectional imaging are essential to establish the diagnosis and guide management [1,5].

Importance of imaging in diagnosis

Imaging plays a critical role in diagnosing and managing SMH because clinical presentation and laboratory findings are often nonspecific. Computed tomography (CT), particularly contrast-enhanced imaging, is the preferred diagnostic modality because it can identify hematoma location, assess lesion size, detect active bleeding, and evaluate surrounding structures [9]. Serial imaging may also help differentiate hematomas from abscesses, inflammatory masses, or neoplastic processes and guide decisions about conservative versus interventional management [9,10].

Significance of this case report

This case is notable because severe bronchitis-associated coughing may have contributed to hematoma formation in the setting of dual antiplatelet therapy and underlying vascular risk factors. The coughing episode may have generated sufficient increases in intra-abdominal pressure to contribute to mesenteric vessel injury and hematoma formation. A literature review indicates that coughing is a very rare precipitating factor for mesenteric hematoma formation. Furthermore, during the initial workup, it was thought that the patient had complicated diverticulitis, illustrating the diagnostic challenge posed by SMH, which frequently mimics more common abdominal emergencies such as appendicitis, diverticulitis, bowel ischemia, perforation, or obstruction.

## Case presentation

Patient demographics and past medical history

A 67-year-old man with a past medical history of hypertension, hyperlipidemia, right inguinal hernia, and coronary artery disease status post four-vessel coronary artery bypass grafting x4 was brought from home by emergency medical services after calling 911 for abdominal pain that had been worsening for five hours prior to arrival. The pain began the previous day as diffuse abdominal pain and later localized to the suprapubic/pelvic region. The pain worsened with bearing down and defecation. During the initial assessment, he reported associated nausea, diaphoresis, and dizziness without vomiting. He reported that he had experienced a severe cold with a severe coughing episode approximately two weeks prior, after which he developed persistent mild lower abdominal pain. He denied fever, shortness of breath, chest pain, jaw pain, epigastric pain, or dysuria. His last bowel movement was the day prior, and he was passing flatus. He reported no prior episodes of similar symptoms. The last colonoscopy performed approximately four to five years earlier was normal. He had no significant family history and no known allergies. He denied smoking and alcohol use. On admission, his home medications included aspirin 81 mg PO once daily, atorvastatin calcium 40 mg PO at bedtime, brimonidine/dorzolamide 0.15%/2% ophthalmic drops twice daily, clopidogrel bisulfate 75 mg PO once daily, famotidine 40 mg PO at bedtime, metoprolol tartrate 25 mg twice daily, pantoprazole sodium 40 mg PO once daily, sertraline hydrochloride 50 mg PO once daily, and timolol maleate 0.5% ophthalmic drops once daily. The patient's initial vital signs were blood pressure 132/80 mmHg, heart rate 70 beats/min, respiratory rate 17 breaths/min, temperature 97.4°F, oxygen saturation 97%, height 182.88 cm, weight 124.738 kg, and BMI 37.3 kg/m2. Initial physical examination showed the patient in no acute distress and appearing well. His skin was warm and dry, with no cyanosis or rash. The head was normocephalic and atraumatic, with no swelling or tenderness. The eyes showed no conjunctival pallor or scleral icterus. The airway was patent without stridor. No cervical lymphadenopathy was noted. Respiratory examination revealed normal bilateral breath sounds without wheezes, rhonchi, or rales. Cardiovascular examination showed a regular rate and rhythm with no murmur or gallop. The abdomen was soft and nondistended. However, he had tenderness in the lower quadrants of the abdomen without guarding or rigidity. A right inguinal hernia extending into the scrotum was noted; it was soft and reducible. Musculoskeletal examination showed no joint swelling or deformity. Bilateral 1+ pitting edema was present, with 2+ pulses in all extremities. Neurological examination showed that the patient was alert, awake, and oriented. Sensation was grossly intact throughout, and he demonstrated full strength in all extremities.

Table 1 summarizes all laboratory findings obtained during the patient's hospitalization.

**Table 1:** Laboratory findings WBC: white blood cells, RBC: red blood cells, eGFR: estimated glomerular filtration rate, AST: aspartate aminotransferase, ALT: alanine aminotransferase

Parameter	Day 0/23:50	Day 1/02:45	Day 1/03:17	Day 2/05:07	Day 3/06:31	Day 4/08:09	Units	Reference range
WBC	11.0	-	-	9.1	6.7	6.7	K/uL	4.5-11.0
RBC	4.43	-	-	3.80	3.46	3.64	M/uL	4.50-5.90
Hemoglobin	13.0	-	-	11.3	10.2	10.5	g/dL	13.5-17.5
Hematocrit	40.2	-	-	35.2	32.2	33.7	%	41.0-53.0
Mean corpuscular volume	90.7	-	-	92.6	93.1	92.6	fL	80.0-100.0
Mean corpuscular hemoglobin	29.3	-	-	29.7	29.4	28.8	pg	27.0-33.0
Mean corpuscular hemoglobin concentration	32.3	-	-	32.1	31.7	31.2	g/dL	32.0-36.0
Red cell distribution width	12.6	-	-	12.9	12.8	12.5	%	11.0-15.0
Platelet count	231	-	-	205	179	209	K/uL	150-450
Mean platelet volume	9.3	-	-	9.4	9.7	9.6	fL	7.0-11.0
Immature granulocytes %	0.5	-	-	0.2	0.3	0.4	%	0-1
Neutrophils %	80.3	-	-	76.0	63.2	61.5	%	30-71
Lymphocytes %	9.8	-	-	14.0	24.9	26.0	%	23-54
Monocytes %	7.8	-	-	8.5	10.3	10.4	%	7-15
Eosinophils %	1.1	-	-	1.0	0.9	1.3	%	0-6
Basophils %	0.5	-	-	0.3	0.4	0.4	%	0-1
Absolute immature granulocyte	0.05	-	-	0.02	0.02	0.03	K/uL	0.0-0.1
Absolute neutrophils	8.88	-	-	6.88	4.22	4.14	K/uL	1.8-6.5
Absolute lymphocytes	1.1	-	-	1.3	1.7	1.8	K/uL	1.2-3.9
Absolute monocytes	0.9	-	-	0.8	0.7	0.7	K/uL	0.5-1.2
Absolute eosinophils	0.1	-	-	0.1	0.1	0.1	K/uL	0.0-0.5
Absolute basophils	0.1	-	-	0.0	0.0	0.0	K/uL	0.0-0.1
Nucleated RBC %	0	-	-	0	0	0	%	0.0-0.2
Sodium	140	-	-	140	140	-	mmol/L	136-145
Potassium	4.5	-	-	4.3	3.8	-	mmol/L	3.5- 5.1
Chloride	102	-	-	100	99	-	mmol/L	98-107
Carbon dioxide	28	-	-	29	30	-	mmol/L	20-31
Anion gap	16	-	-	16	16	-	mmol/L	10-20
Blood urea nitrogen	20	-	-	30	16	-	mg/dL	7-18
Creatinine	1.27	-	-	1.53	0.98	-	mg/dL	0.70-1.30
eGFR	57	-	-	46	76	-	mL/min	>60
Glucose	164	-	-	115	90	-	mg/dL	74-106
Lactic acid	-	-	1.5	-	-	-	mmol/L	0.50-2.20
Calcium	9.1	-	-	9.0	8.6	-	mg/dL	8.3-10.6
Phosphorus	-	-	-	5.2	3.3	-	mg/dL	2.4-5.1
Magnesium	1.98	-	-	2.16	1.92	-	mg/dL	1.60-2.60
Total bilirubin	0.9	-	-	1.2	-	-	mg/dL	0.2-1.0
AST	25	-	-	21	-	-	U/L	<34
ALT	27	-	-	23	-	-	U/L	10-49
AST/ALT ratio	0.9	-	-	0.9	-	-	-	0-1.0
Alkaline phosphatase	85	-	-	80	-	-	U/L	46-116
Lipase	34	-	-	-	-	-	U/L	12-53
High-sensitivity troponin I	9.1	-	-	-	-	-	ng/L	0-54
B-natriuretic peptide	39	-	-	-	-	-	pg/mL	<100
Total protein	-	-	-	6.9	-	-	g/dL	5.7-8.2
Albumin	-	-	-	4.2	-	-	g/dL	3.2-4.8
Prothrombin time	13.7	-	-	14.6	-	-	seconds	12-14.8
International normalized ratio	1.09	-	-	1.18	-	-	-	0.9-1.12
Activated partial thromboplastin time	27.6	-	-	37.2	-	-	seconds	22.8-36.5
Urine color	-	Yellow	-	-	-	-	-	Yellow
Urine clarity	-	Clear	-	-	-	-	-	Clear
Urine pH	-	5.5	-	-	-	-	-	4.5-8
Urine specific gravity	-	>1.035	-	-	-	-	-	1.005-1.030
Urine protein	-	Trace A	-	-	-	-	mg/dL	Negative
Urine glucose	-	Negative	-	-	-	-	mg/dL	Negative
Urine ketones	-	Negative	-	-	-	-	mg/dL	Negative
Urine blood	-	Negative	-	-	-	-	-	Negative
Urine nitrate	-	Negative	-	-	-	-	-	Negative
Urine bilirubin	-	Negative	-	-	-	-	-	Negative
Urine urobilinogen	-	1.0	-	-	-	-	EU/dl	0.2-1.0
Urine leukocyte esterase	-	Negative	-	-	-	-	-	Negative
Urine casts	-	0-2	-	-	-	-	LPF	0-2
Urine epthel cells	-	0-2	-	-	-	-	HPF	0-2
Urine bacteria	-	None seen	-	-	-	-	-	None seen
Urine RBC	-	0-2	-	-	-	-	HPF	0-2
Urine WBC	-	0-5	-	-	-	-	HPF	0-2

Imaging studies were obtained in the following sequence: (1) CT abdomen and pelvis with intravenous (IV) contrast (Figures 1-2), (2) single-view chest X-ray (Figure 3), (3) CT abdomen and pelvis with PO contrast (Figure 4), and (4) CT angiography abdomen and pelvis (Figure 5).

**Figure 1:**
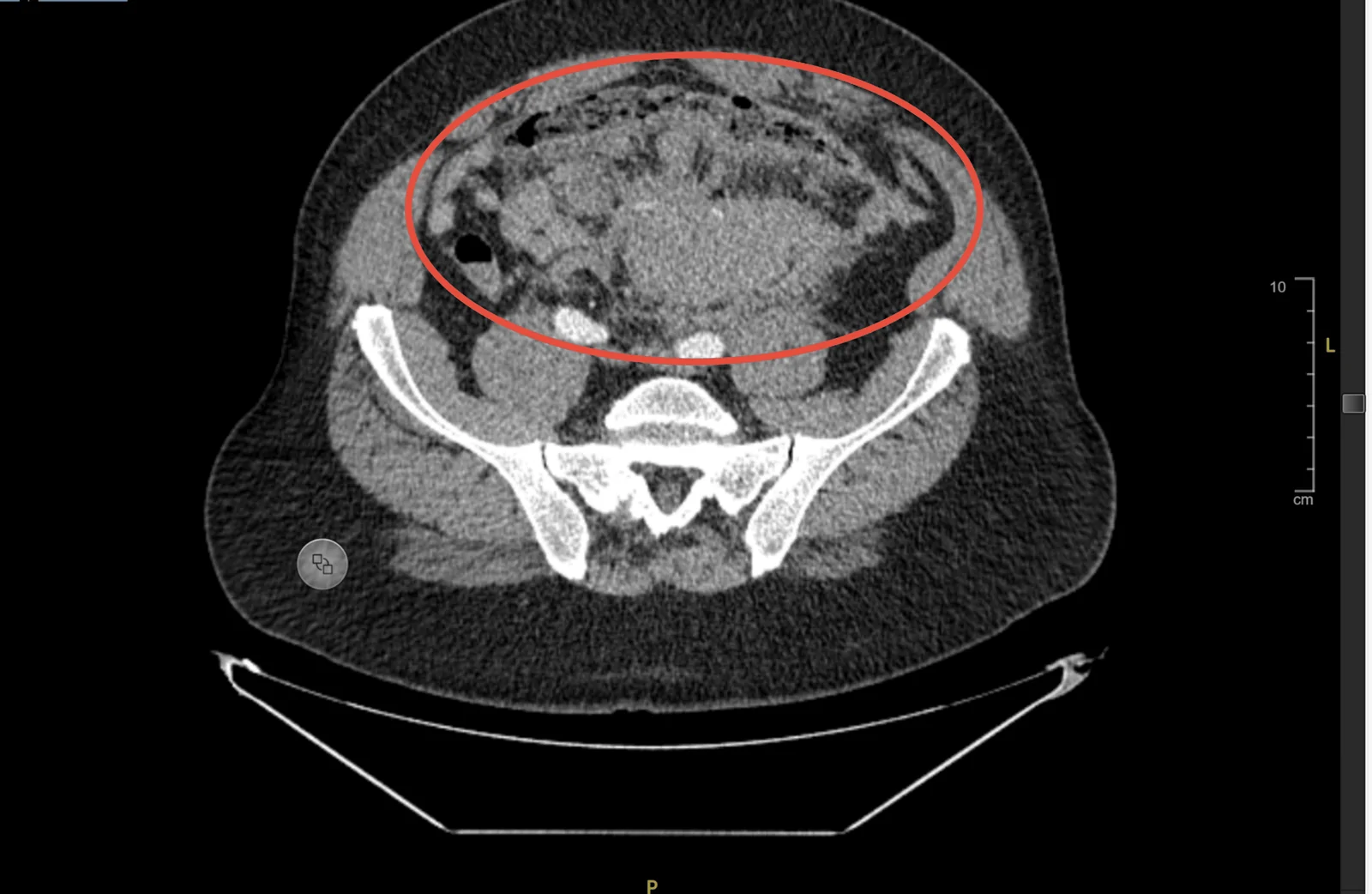
CT abdomen and pelvis with IV contrast Initial findings were interpreted as acute complicated sigmoid diverticulitis with significant surrounding fat stranding and a large focal abscess collection/infected hematoma. The collection contained a hyperdense vessel within the region of interest, raising concern for an infected hematoma. There was edema and pelvic fluid surrounding the sigmoid loop of bowel, as well as fluid in the right lower quadrant, without evidence of bowel obstruction. See Figure 2 for more findings. CT: computed tomography, IV: intravenous

**Figure 2:**
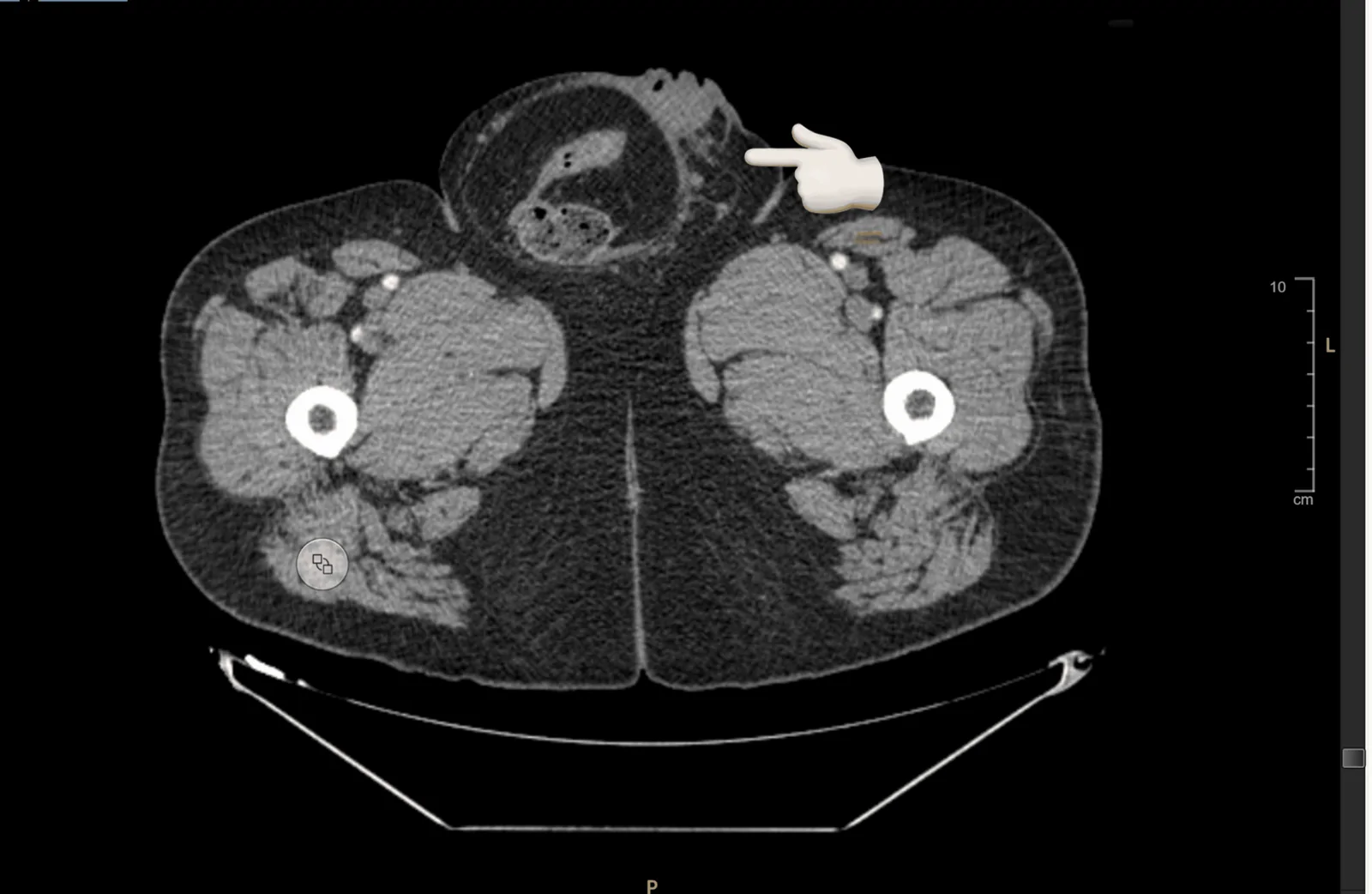
CT abdomen and pelvis with IV contrast Findings demonstrated a large right-sided inguinal hernia containing a loop of sigmoid colon distal to the area of acute diverticulitis. CT: computed tomography, IV: intravenous

**Figure 3:**
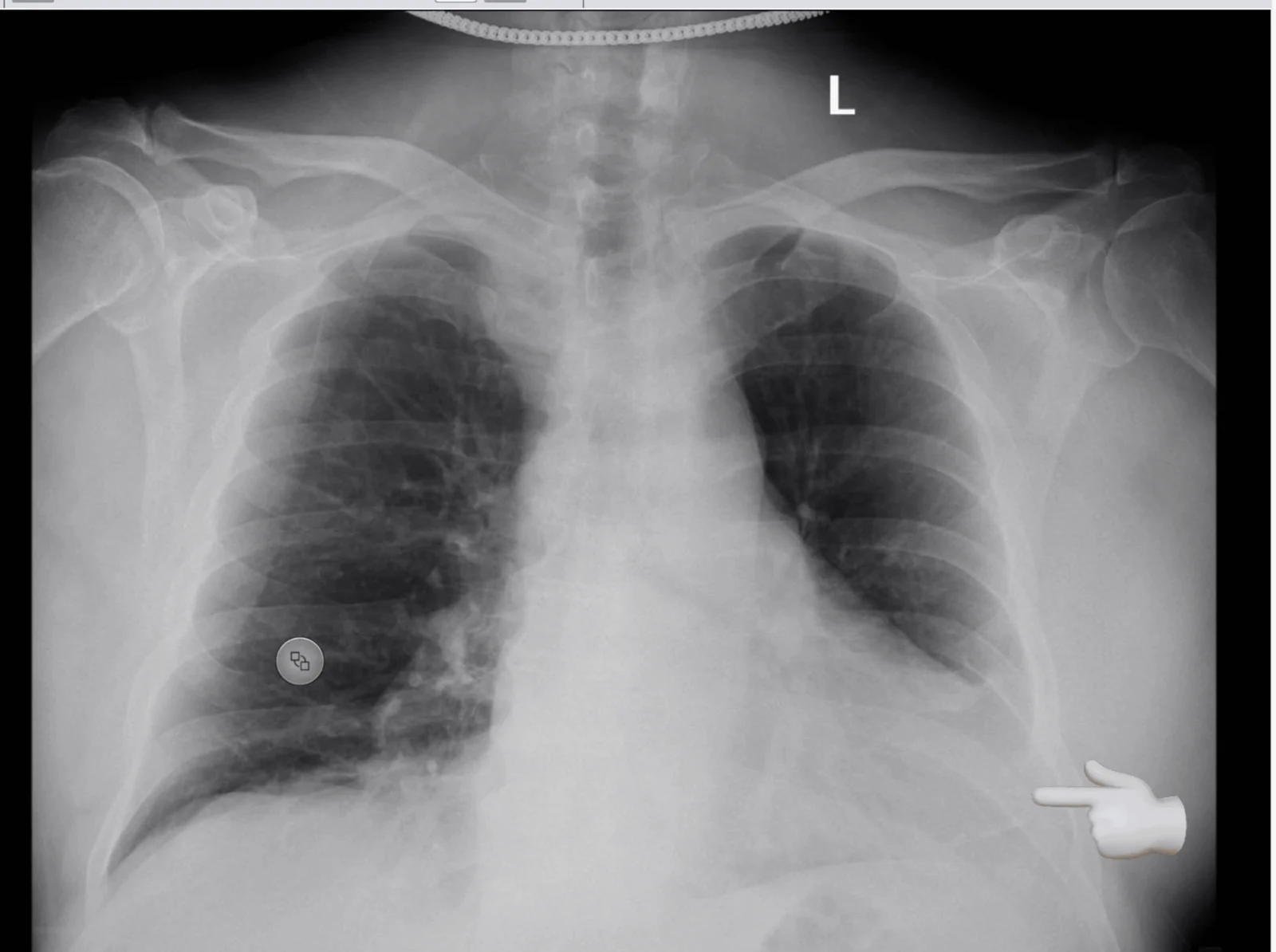
Single-view chest X-ray A possible left-sided pleural effusion was noted.

**Figure 4:**
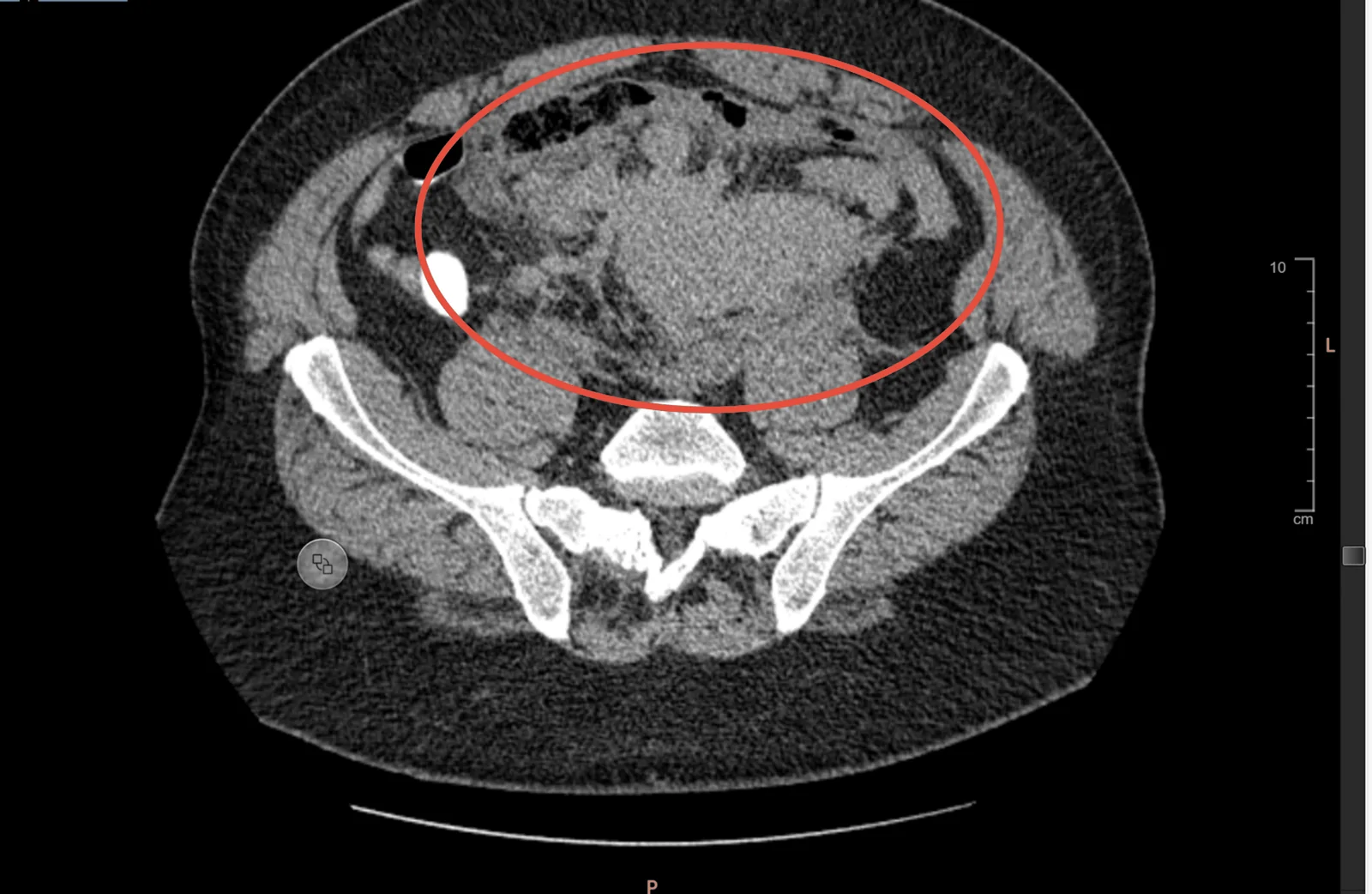
CT abdomen and pelvis with PO contrast Evaluation was limited by the absence of IV contrast. A left lower abdominal collection demonstrated minimal interval enlargement compared with the prior study, with surrounding inflammatory changes. The collection was most consistent with a hematoma. This noncontrast examination limited evaluation for active extravasation. CT angiography was recommended for further evaluation. CT: computed tomography, IV: intravenous, PO: per os

**Figure 5:**
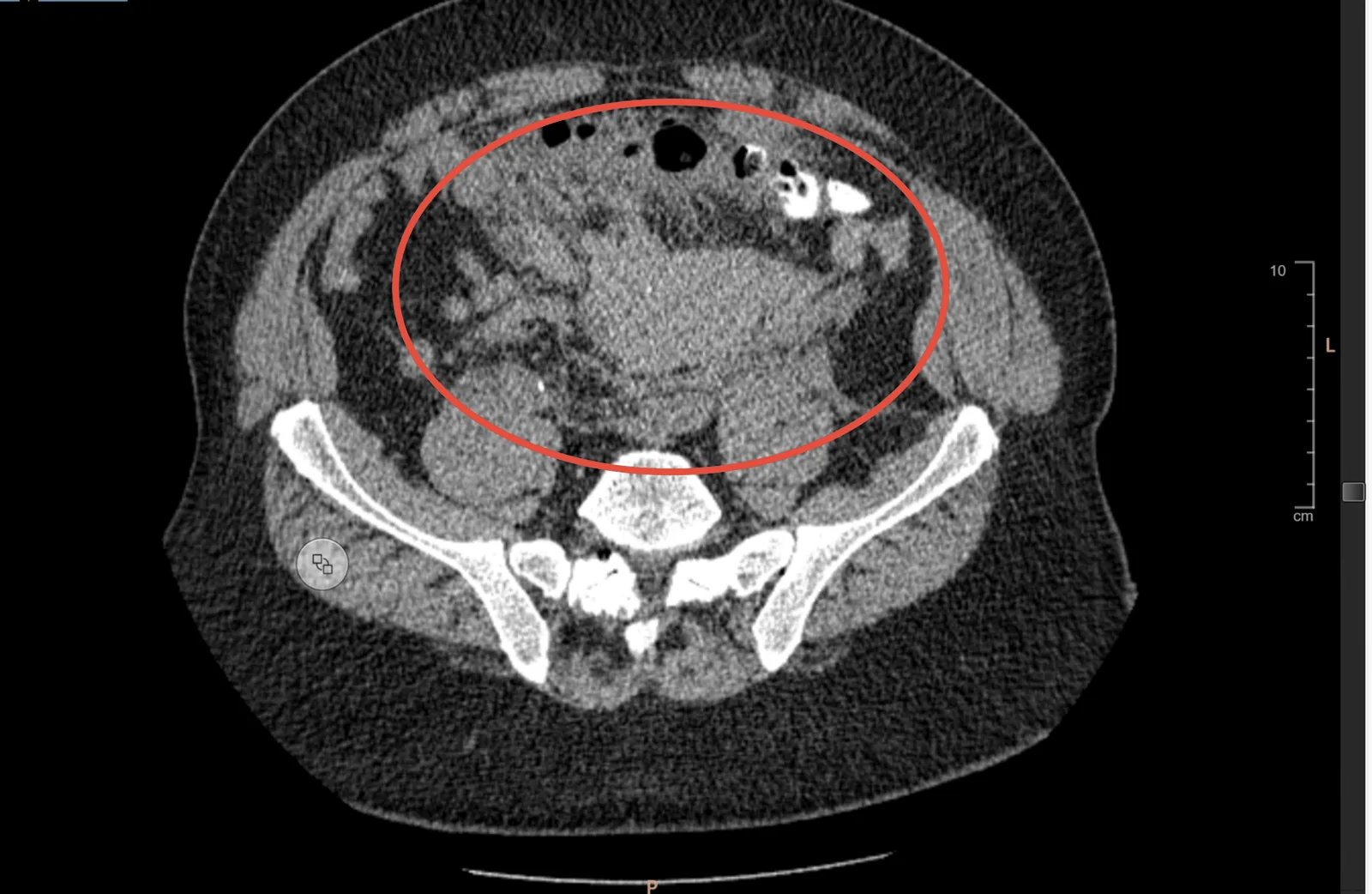
CT angiography abdomen and pelvis Findings showed that the left lower abdominal collection (most likely a hematoma) was unchanged compared with the examination performed earlier that day. Multiphase imaging showed no evidence of active contrast extravasation. A small volume of ascites was noted and remained similar to the prior study. CT: computed tomography

Hospital course

After the patient arrived in the emergency department (ED), he underwent an initial assessment. Given his abdominal pain and concerning physical examination findings, a CT abdomen and pelvis with IV contrast was obtained, revealing a large inflamed fluid collection adjacent to the sigmoid colon, raising concern for an abscess versus hematoma in the setting of suspected complicated diverticulitis. A chest X-ray showed a possible left-sided pleural effusion.

While in the ED, the patient was started on ondansetron hydrochloride 4 mg IV for nausea, piperacillin/tazobactam 3.375 mg IV for possible infection, and morphine sulfate 4 mg IV push for pain control. The surgical team subsequently evaluated him and admitted him for further management and monitoring.

The surgical team discussed the case with interventional radiology (IR), and a repeat CT abdomen and pelvis with PO contrast was obtained to better characterize the abdominal collection. The patient was kept NPO for bowel rest and possible intervention. Given the concern for infection versus hematoma, he was continued on piperacillin/tazobactam 3.375 mg IV every six hours. Additional inpatient management included pain control with morphine sulfate and acetaminophen, enoxaparin for deep vein thrombosis (DVT) prophylaxis, and pantoprazole for stress ulcer prophylaxis.

A repeat CT abdomen and pelvis with PO contrast demonstrated a left lower abdominal collection with minimal interval enlargement compared with the prior study and persistent inflammatory changes, favoring a hematoma rather than an abscess. Evaluation for active extravasation was limited because IV contrast was not obtained, and CT angiography was recommended. Given the evolving imaging findings and concerns that the collection represented a hematoma rather than an infectious process, the infectious disease (ID) service was consulted and recommended monitoring of the white blood cell count and temperature curve.

Given the concern for an intra-abdominal hematoma, the patient's home aspirin 81 mg daily and clopidogrel 75 mg daily were held. Enoxaparin 40 mg subcutaneously was initially administered for DVT prophylaxis. It was subsequently changed to unfractionated heparin 5,000 units subcutaneously every eight hours on hospital day 2 in the setting of acute kidney injury.

At the end of hospital day 1, IR and the surgical team proceeded with CT angiography of the abdomen and pelvis to evaluate for active bleeding. CT angiography demonstrated an unchanged left lower abdominal collection, favored to represent a hematoma, without evidence of active contrast extravasation on multiphase imaging. IR determined that hematoma drainage was not necessary due to its stability and the patient’s hemodynamic stability. The findings supported a diagnosis of SMH, which initially mimicked complicated diverticulitis with abscess formation on presentation. Conservative management was recommended with serial hemoglobin monitoring.

On hospital day 2, the patient remained clinically stable with improving abdominal pain. He was encouraged to ambulate and increase activity as tolerated. After discussion with the ID service, piperacillin/tazobactam was discontinued; the last dose was administered that morning. The patient continued to be monitored closely for any signs of infection, including changes in white blood cell count, temperature trends, or any worsening abdominal pain.

On hospital day 3, the patient was advanced to a regular diet and continued to improve clinically, with well-controlled pain and resolving abdominal symptoms. He had a bowel movement overnight but continued to report constipation. Docusate sodium 100 mg PO every 12 hours as needed was added for constipation. IR was reconsulted regarding possible hematoma drainage and continued to recommend conservative management given the stable imaging findings. The patient was advised to undergo follow-up colonoscopy after complete resolution of the acute process.

On hospital day 4, the patient was evaluated by ID and cleared for discharge without the need for antibiotic therapy. Given his clinical improvement, stable hemoglobin, controlled symptoms, and ability to tolerate oral intake, he was deemed medically stable for discharge.

## Discussion

Overview of SMH

SMH is an underdiagnosed cause of acute abdominal pain. SMH is localized bleeding into the mesentery or bowel wall without preceding trauma, typically associated with prolonged antithrombotic therapy, including antiplatelet agents or anticoagulants (most commonly warfarin), bleeding disorders (hemophilia, thrombocytopenia), vascular fragility (hypertension, atherosclerosis), and malignancy [4,8]. In the absence of these etiologies, mesenteric hematoma is considered idiopathic, indicating that no apparent underlying cause has been identified [4,8].

SMH is rare, with approximately 110 reported cases in the National Library of Medicine database [4] since its first description in the early 1900s. Proposed causes include trauma, connective tissue disorders (e.g., Ehlers-Danlos syndrome), coagulopathies, and arteriopathies. The reported cases of SMH emphasize three recurring and recognizable clinical patterns: anticoagulation-associated hemorrhage, idiopathic presentations, and complicated cases requiring surgical intervention [4].

The clinical presentation of SMH ranges from benign, self-limited collections to life-threatening hemorrhage. Management depends on hemodynamic stability, anticoagulation status, radiologic evidence of active bleeding, and bowel compromise. Conservatively managed cases have been documented without underlying pathology. Spontaneous resolution with close radiologic surveillance has been reported in hemodynamically stable patients without evidence of ongoing hemorrhage, as demonstrated in this case. Surgical intervention is typically reserved for cases with diagnostic uncertainty or complications [1,4,5,8].

Pathophysiology of cough-induced mesenteric vessel injury

The mechanism of SMH following severe coughing is not fully understood but is believed to involve abrupt elevations in intra-abdominal pressure, causing stress and rupture of small mesenteric vessels. Similar mechanisms have been described in cough-associated rectus sheath hematomas and other spontaneous intra-abdominal bleeding events. Repetitive, forceful coughing may generate sufficient mechanical stress to injure fragile vessels, particularly in patients with additional predisposing factors such as advanced age, vascular disease, obesity, or antiplatelet or anticoagulant therapy [6,7]. In the present case, the patient reported a severe coughing episode approximately two weeks before symptom onset and was receiving dual antiplatelet therapy with aspirin and clopidogrel, which may have further increased his susceptibility to bleeding.

Differential diagnosis of acute abdominal pain and intra-abdominal hemorrhage

In patients presenting with acute abdominal pain, clinicians must exclude life-threatening differential diagnoses, including vascular emergencies (e.g., rupture of the superior mesenteric, hepatic, or splenic arteries; aortic dissection; or aortic aneurysm), neoplastic conditions (e.g., mesenteric tumors and lymphomas), inflammatory conditions (e.g., inflammatory bowel disease and acute pancreatitis), and systemic causes (e.g., coagulopathies and anticoagulation therapy). CT plays an important role in assessing the presence, location, and extent of hemorrhage and in identifying the underlying cause, which is essential for prompt diagnosis and patient management [11].

The age and location of the hemorrhage on CT imaging influence its appearance. Acute bleeding or clotted blood on noncontrast images appears hyperdense (>60 HU) because its high protein content increases attenuation compared with surrounding lower-attenuation serum [11]. On contrast-enhanced CT, active hemorrhage appears nearly isodense to adjacent vessels, suggesting active bleeding and the potential need for emergency embolization or surgical intervention [11].

Diagnostic imaging findings and role of CT abdomen/pelvis

SMH diagnosis is challenging because of its nonspecific clinical presentation and radiologic similarity to neoplastic or inflammatory masses [8]. Contrast-enhanced CT is the primary diagnostic modality for SMH and plays a critical role in identifying the location, extent, and characteristics of the hematoma. Typical CT findings include a localized hyperdense mesenteric mass with attenuation values that vary with the age of the hemorrhage. In the acute phase, the high protein concentration, intracellular hemoglobin degradation products, and increased viscosity of the hematoma contribute to increased attenuation on CT imaging. As the hematoma evolves, changes in its appearance may mimic other pathological processes, including hypercellular malignant lesions or inflammatory masses, complicating diagnosis [8,11].

Multiphase CT angiography is particularly valuable when active bleeding, pseudoaneurysm formation, or associated bowel ischemia is suspected, as it can demonstrate active contrast extravasation and help guide interventional management. Angiography may further help localize the bleeding source when intervention is required. Ultrasound or magnetic resonance imaging may also serve as supplementary modalities for follow-up or in patients who cannot receive iodinated contrast agents [8].

Conservative versus surgical management

Management of SMH depends largely on hemodynamic stability, evidence of active hemorrhage, and the presence of complications, including bowel ischemia, perforation, or an enlarging hematoma. Hemodynamically stable patients without active contrast extravasation on imaging can frequently be managed conservatively with serial abdominal examinations, laboratory monitoring, and repeat imaging when indicated [2,3]. Surgical exploration or interventional procedures may be reserved for patients with ongoing bleeding, diagnostic uncertainty, or clinical deterioration. In this case, serial imaging demonstrated a stable hematoma without active extravasation, allowing successful conservative management without surgical or interventional radiologic intervention.

Outcomes, prognosis, and follow-up

The prognosis of SMH is generally favorable when the diagnosis is established early and active hemorrhage, bowel ischemia, and hemodynamic instability are absent. In hemodynamically stable patients, conservative management consisting of serial abdominal examinations, laboratory monitoring, and interval imaging has been associated with successful resolution in numerous reported cases. Conversely, delayed diagnosis or progression of hemorrhage may result in bowel compromise, the need for operative intervention, or, rarely, death [1,2,3,9].

In the present case, the patient remained hemodynamically stable throughout hospitalization. Serial imaging showed a stable left lower abdominal hematoma without active contrast extravasation, and hemoglobin decreased from 13.0 g/dL on admission to a nadir of 10.2 g/dL on hospital day 3, then remained stable without evidence of ongoing bleeding. IR determined that drainage was not indicated because the lesion was stable and no drainable collection was present. The patient's abdominal pain gradually improved with supportive care, bowel rest, analgesia, and close observation, allowing discharge without surgical intervention. This clinical course is consistent with previously reported cases demonstrating that conservative management is a safe and effective strategy in carefully selected patients without evidence of ongoing hemorrhage or associated complications [2,3].

Long-term follow-up in patients with SMH should focus on confirming hematoma resolution, reassessing antithrombotic therapy when applicable, and evaluating for occult vascular, hematologic, or neoplastic etiologies when clinically indicated. Colonoscopy after resolution of the acute process may also be considered, particularly when the initial presentation or imaging findings raise concern for underlying colonic pathology.

Review of similar cases in the literature

SMH remains an uncommon diagnosis, with most published reports consisting of isolated case reports and small case series. Reported risk factors include anticoagulant or antiplatelet therapy, coagulopathies, connective tissue disorders, vascular abnormalities, hypertension, and other conditions associated with vascular fragility, although many cases remain idiopathic despite extensive evaluation [1,4,5].

Several reports have described SMH in patients receiving antithrombotic therapy. Phua et al. reported cases occurring in patients receiving novel oral anticoagulants for atrial fibrillation [4]. Aoki et al. described a patient receiving rivaroxaban 15 mg daily and cilostazol 200 mg daily who developed SMH requiring surgery because of persistent symptoms and diagnostic uncertainty [5]. Hirano et al. reported a 57-year-old woman receiving aspirin therapy for idiopathic thrombocytosis who developed an SMH adjacent to the sigmoid colon [12].

The anatomic location of the hematoma in our patient is also notable. Most reported SMHs involve the small bowel mesentery, particularly the jejunal or ileal mesentery, whereas a left lower abdominal hematoma adjacent to the sigmoid colon is less frequently described [3,5,12]. Similar to the case reported by Hirano et al., our patient's hematoma was located near the sigmoid colon. This location may complicate diagnosis because surrounding inflammatory changes can mimic diverticulitis, abscess formation, or colonic malignancy [12].

Our patient shared several features with previously reported cases, including antithrombotic exposure and vascular risk factors. He had hypertension, hyperlipidemia, obesity, extensive coronary artery disease with a history of coronary artery bypass grafting, and chronic dual antiplatelet therapy with aspirin 81 mg daily and clopidogrel 75 mg daily. Unlike many cases involving therapeutic anticoagulation, however, our patient was receiving dual antiplatelet therapy alone. Initial imaging favored complicated diverticulitis with abscess formation, but subsequent studies showed a stable mesenteric hematoma, further illustrating the diagnostic uncertainty described in the literature [1,4,5].

Another distinguishing feature was a history of severe bronchitis-associated coughing about two weeks before symptom onset. Prior reports of cough-related abdominal hemorrhage, including rectus sheath hematomas, suggest that repetitive increases in intra-abdominal pressure may contribute to the rupture of susceptible vessels [6,7]. Carey et al. reported acute mesenteric hematoma and hemoperitoneum following a prolonged coughing episode in a patient with COVID-19 infection who was not receiving anticoagulant therapy [13]. The authors proposed that coughing-related mechanical stress and COVID-19-associated vascular injury may have both contributed to hemorrhage. Similarly, in our patient, severe coughing may have acted in concert with underlying vascular disease and dual antiplatelet therapy to increase susceptibility to mesenteric bleeding [13].

To our knowledge, reports specifically linking bronchitis-associated coughing to SMH remain exceedingly uncommon. This case adds to the limited literature suggesting that severe coughing may represent an underrecognized precipitating factor in patients with concurrent vascular and antithrombotic risk factors.

Clinical importance and learning points

This case highlights several important learning points. First, SMH may occur in the absence of trauma, therapeutic anticoagulation, or a known bleeding disorder. Second, antiplatelet therapy and underlying vascular risk factors, including hypertension, hyperlipidemia, obesity, and atherosclerotic cardiovascular disease, may contribute to hemorrhage even when routine coagulation studies are normal. Third, the location of the hematoma adjacent to the sigmoid colon raised concern for complicated diverticulitis, showing how anatomic location can influence diagnostic interpretation and potentially delay recognition of the underlying condition.

Finally, clinicians should maintain a high index of suspicion for SMH in patients presenting with atraumatic abdominal pain, particularly when imaging findings are atypical or fail to fit a single unifying diagnosis. A thorough history should include recent episodes of severe coughing, vomiting, heavy lifting, constipation, or other events associated with marked increases in intra-abdominal pressure. In this patient, the temporal relationship between severe bronchitis-associated coughing and symptom onset raises the possibility that coughing precipitated the event in the setting of underlying vascular vulnerability and dual antiplatelet therapy. Recognizing these factors may enable earlier diagnosis, prevent unnecessary invasive procedures, and support successful conservative management in appropriately selected patients.

## Conclusions

SMH is a rare and potentially challenging diagnosis due to its nonspecific presentation and ability to mimic other causes of acute abdominal pain. Severe coughing may represent an uncommon precipitating factor through repeated increases in intra-abdominal pressure, particularly in patients with additional bleeding risk factors such as antiplatelet therapy. Prompt recognition and appropriate imaging are essential for diagnosis, while stable patients may be successfully managed with conservative treatment strategies. This case highlights the importance of considering SMH in patients presenting with abdominal pain after severe coughing episodes.
